# [Retracted] Dapagliflozin can alleviate renal fibrosis in rats with streptozotocin-induced type 2 diabetes mellitus

**DOI:** 10.3892/etm.2026.13244

**Published:** 2026-07-20

**Authors:** Song Xue, Ying-Xuan Li, Xiao-Xiao Lu, Wei Tang

Exp Ther Med 26:572, 2023; DOI: 10.3892/etm.2023.12271

Following the publication of the above paper, the authors requested that a corrigendum be published after they had identified that errors were made in assembling the western blot data in Figs. 4E and 5B. Upon performing an independent analysis of the data in this paper, however, it came to light that the vimentin blots in Fig. 4E and the Total-Smad3 blots in Fig. 5B were strikingly similar to data that had already appeared in other articles written by different authors at different research institutes published in the journals *Cancer Medicine Reports* and *Inflammation* respectively.

Given that the contentious data described above had already been published before this paper was submitted to *Experimental and Therapeutic Medicine*, the Editor has decided that this paper should be retracted from the Journal. After contacting the authors, they accepted the decision to retract the paper. The Editor apologizes to the readership for any inconvenience caused.

